# Platelet-Rich Plasma, Hydroxyapatite, and Chitosan in the Bone and Cartilaginous Regeneration of Femoral Trochlea in Rabbits: Clinical, Radiographic, and Histomorphometric Evaluations

**DOI:** 10.1155/2018/6917958

**Published:** 2018-06-24

**Authors:** Francisco Alipio de Sousa Segundo, Edla Iris de Sousa Costa, Adílio Santos de Azevedo, Ana Lucélia de Araújo, Ana Clara de França Silva, Gabriel Goetten de Lima, Marcelo Jorge Cavalcanti de Sá

**Affiliations:** ^1^Federal Institute of Paraíba, IFPB, João Pessoa, PB, Brazil; ^2^Veterinary Hospital, Patos Campus, Federal University of Campina Grande (UFCG), Campina Grande, PB, Brazil; ^3^Materials Research Institute, Athlone Institute of Technology (AIT), Athlone, Ireland

## Abstract

The aim of this study was to evaluate the trochlear bone and cartilaginous regeneration of rabbits using the association of PRP, chitosan, and hydroxyapatite. Hole was made in rabbit troches, one hole in each animal remained empty (group C), and one was filled by a combination of PRP, chitosan, and hydroxyapatite (group T). Clinical-orthopedic, radiographic, and histomorphometric evaluations were performed. Clinical-orthopedic evaluation showed lameness of two members of the T group and one member of group C. The radiographic evaluation showed that the T group showed absence of subchondral bone reaction (33%). The presence of moderate subchondral bone reaction was more frequently reported in group C with 67%. Microscopic evaluation revealed a presence of tissue neoformation, composed of connective tissue. Microscopic findings were similar in both groups, with a difference in the amount of neoformed tissue being perceptible, which was confirmed after the morphometric analysis, which revealed a significant difference in the quantity of newly formed tissue at the bone/cartilage/implant interface. The composite base of the association of chitosan, hydroxyapatite, and platelet-rich plasma favored bone and cartilage healing.

## 1. Introduction

Cartilaginous tissue is mainly present in the joints, and it consists predominantly of type II collagen and glycoproteins, which promote the tissue functions of supporting biomechanical forces, such as tension and compression, generated during the process of ambulation [1–3].

The articular cartilage has a very limited regenerating or healing capacity in lesions with no involvement of the subchondral bone, resulting in an empty lacuna. This characteristic of the tissue causes traumas or degenerative diseases in this region, which are difficult to solve [4–6].

The current treatments for regeneration of the articular cartilages may be conservative or surgical, but they are not very successful, since the damaged tissue is replaced by fibrous tissue or fibrocartilage, which present inferior biomechanical functions to the cartilaginous tissue [7–9].

In recent years, the use of cellular therapies and the use of biomaterials to assist the healing process in different tissues, including injured joint cartilage, has been increasing [10, 11]. Among these, the use of chitosan, which is a polymeric biomaterial, has been highlighted by its power to stimulate the healing and regeneration of several organic tissues [12–14].

Hydroxyapatite, classified as calcium phosphate cement, is reported as a widely used biomaterial, mainly in bone lesions due to its physicochemical structure similar to the bone, serving as a component for osteoconduction and bone repair processes [15, 16].

As well as chitosan, platelet-rich plasma (PRP) is another alternative with regard to tissue regenerative therapies, being easy to obtain, and it also presents peptide components known as inducing factors, which act in the proinflammatory process, triggering the repair in several tissues [17–19].

The association of PRP, chitosan, and hydroxyapatite in the healing of bone or cartilaginous tissue has not been reported in literature. The aim of the present study was to evaluate the trochlear bone and cartilaginous regeneration of rabbits by the association of PRP, chitosan and hydroxyapatite.

## 2. Materials and Methods

### 2.1. Sample Preparation

Twelve adult rabbits, New Zealand, weighing on average 1.7 ± 0.5 kg were used. For this study, the knees of the rabbits were investigated where one of the knees was left untreated and the other knee was treated with a composition of PRP, chitosan, and hydroxyapatite, so two groups were formed:

- Group C: the control group, which had a circular bone hole in the middle of the right femoral trochlea, with 4 mm diameter and 3 mm depth, where it was left untreated.
- Group T: the treatment group, in which a bone hole of the same size was made, but in the left femoral trochlea; PRP associated with chitosan and hydroxyapatite (PRP + Q + H) was implanted in the lesion.

The animals underwent an adaptation period of 21 days before the start of the experiment and were treated with 10% albendazole at a dose of 20 mg/kg live weight orally. During the trial period, the animals were housed in cages, in a protected environment from sun and rain, received commercial balanced ration for rabbits twice a day, and had access to drinking water at will.

### 2.2. Production of Chitosan

The polymeric solution was prepared by dissolving 3 g of chitosan in 100 ml of a 1% (v/v) glacial acetic acid under magnetic stirring at 45°C for 2 h. This solution was poured into Petri dishes and packed in an ultrafreezer at −70°C for 24 h where this material was lyophilized for 72 h. After freeze-drying, the sieves were immersed in 1 M sodium hydroxide for 1 h and washed with distilled water to remove excess sodium hydroxide. This solution was again immersed in distilled water, frozen in an ultrafreezer, and freeze-dried to obtain the scaffolds with pH close to neutral.

### 2.3. Production of Hydroxyapatite

Calcium phosphate was obtained through a wet precipitation method involving a neutralization reaction between solutions of phosphoric acid (H_3_PO_4_) and calcium hydroxide [Ca(OH)_2_]. The quantities of the solutions were stoichiometrically determined according to the value of the atomic ratio between the calcium and phosphorus atoms. The Ca(OH)_2_ powder was added to deionized water, stirred, and heated to a temperature of 80°C. This solution was slowly added the H_3_PO_4_ solution under constant stirring. After complete mixing of the two reactants, the temperature was raised to 100°C and stirring was maintained until the viscosity of a paste was reached. The obtained ceramic paste was dried at 110°C for 24 hours, and the product was deagglomerated and graded to 200 meshes until it was possible to obtain a powder; this was further heat treated at 20°C/min and held at 1100°C for 2 hours.

### 2.4. Production of Platelet-Rich Plasma (PRP)

The PRP was performed according to the protocol of Wu et al. [20]. Immediately before the operative procedure, 3 ml of blood was collected punctured from the jugular vein of each rabbit, and the blood was transferred to a test tube containing ethylenediaminetetraacetic acid (EDTA). A sample of the collected blood was submitted to plaquetogram, and the remaining was subjected to a first centrifugation for 10 minutes at 1800 rpm. Following the protocol, the supernatant plasma obtained from the first centrifugation was transferred to another sterile test tube and centrifuged for an additional 10 minutes in a rotation of 3600 rpm. A sample of the PRP that was located in the lower portion of the test tube was punctured with the aid of a sterile needle and submitted to plaquetogram, and the remaining was applied to the trochlear gap of the T group.

### 2.5. Surgery Procedure

The research project was evaluated and approved by the Research Ethics Committee of the Federal University of Campina Grande (UFCG) number 072/2017. In the preoperative period, each animal was fasted from solid for 6 hours and liquid for 2 hours. The animals had both clipped limbs, with a wide margin for the operative field. The animals were pretreated to the operative procedure with antibiotic prophylactic therapy using ceftriaxone 20% at the dose of 30 mg/kg administered 30 minutes before the operation, in order to avoid the infectious process. For pain control, preemptive analgesia, to avoid an exacerbated inflammatory process, was administered by the intramuscular (IM) route with 30 minutes before the surgical procedure by the use of meloxicam at the dose of 0.2 mg/kg, followed on the next two days using 0.1 mg/kg. Preanesthetic medication consisted of 1% acepromazine at a dose of 0.2 mg/kg IM and anesthesia with tiletamine associated with 10% zolazepam at a dose of 15 mg/kg (IM). Lumbosacral epidural anesthesia was also performed in the total dose of 0.3 ml/kg, with a 2% lidocaine combination with 2% xylazine and 1% morphine at doses of 0.22 ml/kg, 1, 5 mg/kg, and 0.1 mg/kg, respectively.

The antisepsis of the operative areas was performed with 0.5% chlorhexidine alcohol solution. After delimitation of the operative area with field cloths, in group C, a lateral parapatellar cutaneous incision of the right knee was performed, followed by arthrotomy with joint exposure. Following the procedure, a circular bone hole with trephine measuring 4 mm in diameter and 3 mm in length was made. After the preparation of the orifice and withdrawal of the cap, the capsulorrhaphy was performed with separated “Wolf” stitches with surgical nylon 2-0. The subcutaneous space was reduced by using 3-0 chromic catgut with intradermal suture pattern and dermorrhaphy using 3-0 surgical nylon and separate “Wolf” suture pattern.

In the T group, the same procedure was performed on the contralateral limb, so the prepared bone hole was filled with chitosan, hydroxyapatite, and PRP, in this order.

In the first ten postoperative days (PO), surgical wound antisepsis with 0.9% physiological solution and a commercial antibacterial spray was used until removal of the external points.

### 2.6. Clinical-Orthopedic Evaluation

The animals were evaluated daily during the first ten days of the PO until the removal of the external surgical points. After this period, the animals were evaluated twice a week throughout the experimental period. The procedure consisted of inspection and palpation of the limbs by evaluating the presence of pain and muscular atrophy of the quadriceps muscle group of the operated limb by comparing with the contralateral limb.

### 2.7. Radiographic Evaluation

Radiographic examinations were performed on the craniocaudal (CC) and lateromedial (LM) projections of both the knees at two moments: 30 days after the surgical procedure and 60 days after the surgical procedure. The evaluation was based on the presence of osteoarthritic lesions, synovial effusion, and the presence of periarticular osteophyte and was compared among themselves, in order to follow the evolution during the experimental period. With the radiographs of 60 days after the surgical procedure, tissue reaction indices were assigned.

### 2.8. Histopathological and Morphometric Evaluation

After 60 days of PO, the animals were slaughtered by dissociation of the cerebral cortex by concussion and subsequent bleeding. The two knees of each animal were evaluated comparatively between themselves and between the groups. After the two hind limbs were removed, the knees were collected and fixed in 10% buffered formalin for 10 days. The knees, after fixation, were decalcified with a solution of 6% hydrochloric acid over a period of 5 days. Then, the fragments were dehydrated by passages in alcohol solutions in increasing concentrations (70%, 80%, 90%, and absolute alcohol). Subsequently, the material was washed in running water, was embedded in liquid paraffin, and subsequently cut into 5 *μ*m thick cross sections comprising the cartilage/bone/bone gap interface filled in or not by treatment according to the experimental group. Following the process, the tissue materials were mounted on glass slides. From each block, four slides were obtained, which were submitted to hematoxylin-eosin techniques. For the histopathological evaluation, repair findings were observed in the existing tissue types (fibrous, fibrocartilage, and/or hyaline cartilage), joint surface regularity, existing cellularity, and tissue adhesion to biomaterials.

The same slides were evaluated by the morphometry process for quantification of the repair tissues (fibrous, fibrocartilage, and/or hyaline cartilage), comparing the groups to each other, through the MvImage program, version 3.1®. To perform this analysis, captures and digitization of the images that comprised the cartilage/bone/lacuna interface were performed. Sequenced images of each lamina analyzed were obtained to quantify all the newly formed tissue in all areas of the cartilage gap. The mean values obtained for each group were obtained and submitted to statistical analysis.

### 2.9. Statistical Analysis

For the comparison of the groups for histomorphometric evaluation, the Kruskal–Wallis test was conducted, with multiple comparisons by the Nemenyi test. The level of significance adopted in all analyses was 5%, and the software BioEstat 5.03 was used.

## 3. Results and Discussion

For the accomplishment of the plaquetograms, the method described by Stockham and Scott [21] was employed, being realized through reading of blood smear blade with staining of Romanowsky (quick panoptic); after this, five fields with well-distributed cellularity was read, and then the mean value of the five fields was multiplied by the factor 20,000 to obtain platelet quantity per mm^3^.

The platelet result of the sample collected before the processing to obtain PRP showed an average value of 250 ± 31.4 thousand platelets/mm^3^, and after processing, a mean increase of 349% ± 11.5 in the platelet values was observed.

The values of platelet concentration found after the processing were in accordance with the one recommended by Marx [22], which reports a greater effectiveness of the platelet concentrate, when it reaches values higher than 1,000,000 platelets/mm^3^.

The results regarding clinical-orthopedic evaluation performed on the animals are described in Table 1.

During the evaluation of the ambulatory process, three animals presented lameness; two of them were the members of group T, and one was the member of group C. The lameness of the animals lasted a period of four days after the surgical procedure, being observed later normality of the walking process.

The evolution of lameness was not observed in the present study, the appearance of the clinical sign was associated with a possible individual response of the animals, and the transient situation did not cause negative clinical influence to the animals.

According to Yamada et al. [23], in a study with intralesional and intra-articular PRP in injured joints of horses, the animals that presented lameness after the surgical procedure of the trauma showed evident clinical improvement after the second PRP application.

The animals affected by lameness in a few days returned to the normal ambulation process, which promoted maintenance of the muscles, which did not suffer any detrimental effects during the experimental period, and suggested that the articular cartilage had the capacity to withstand the biomechanical forces employed.

The lesions of articular cartilage that lead to the appearance of clinical signs are usually progressive affections; that is, they tend to cause a degeneration of the joint, leading to a worsening of the clinical signs and consequent functional loss [24], which was not observed in the present study.

Intra-articular application of platelet-rich plasma is reported as a therapy with high success rates in reducing lameness in horses [25–27]. Clinical-orthopedic evaluation revealed that none of the animals had painful tenderness in any of the limbs. The absence of a painful signal in the limb is probably associated with the use of an anti-inflammatory drug in the correct postoperative period, which prevented the development of joint disease or progressive cartilage damage.

Osteoarthritis can be induced by cartilage damage, although this condition is classified as noninflammatory joint disease with a low-intensity inflammation. During the clinical examination of a patient with this condition, the painful reaction can be observed during palpation, along with other signs such as limitation of amplitude in limb flexion and extension, crepitus, and joint volume increase [28].

Once the limbs of the animals were evaluated, no muscular atrophy was observed when group C and group T members were compared. The quadriceps muscle group atrophy, in the case of joint disease of the pelvic limb, is generally observed as a clinical sign in affected animals and is most often associated with compensatory hypertrophy in other regions [29].

The subchondral bone healing reaction rates attributed to the radiographs obtained 60 days after the surgeries are shown in Table 2.

The T group had a greater number of limbs both with absence of subchondral bone reaction (33%) and presence of intense subchondral bone reaction (25%).

The presence of moderate subchondral bone reaction was more frequently reported in group C with 67% of the limbs described.

Simple radiography is one of the standard imaging tests to identify the presence and progression of articular cartilage lesion and can be used to evaluate the integrity of various joint structures, including bone and cartilage [30].

According to Rasera et al., the radiographic examination has some limitations, and the initial alterations caused by the articular cartilage lesion are only evidenced later, when it progresses and leads to the appearance of secondary bone lesion, demonstrating the dependent relation between the bone and cartilage. The author reports in a study with equine experimental osteoarthritis that radiography was only successful in demonstrating joint cartilage integrity 40 days after the trauma began [31].

The use of radiographs in the present study made it possible to perform an evaluation of the subchondral bone healing reaction and to observe the joint integrity of the limbs, obtaining satisfactory results due to the anatomical relationship between the cartilage and bone.

In addition to providing an anatomical framework for cartilage, the subchondral bone still provides a source of vascularization, and in the case of absent cartilage, this vascularization is necessary for tissue repair to occur [32].

The assignment of indices on the radiographs can be considered a subjective examination, in this way histomorphometric analyses were performed, and the results are expressed in Table 3.

**Figure 1:**
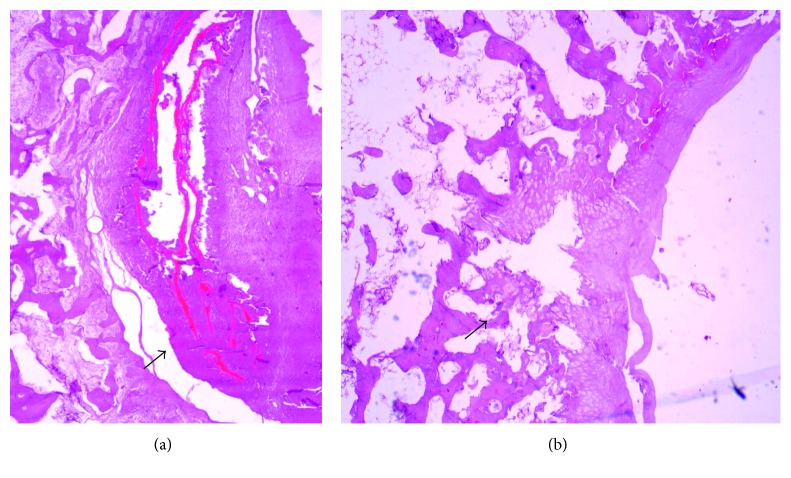
Descriptive histopathological evaluation after 60 days. (a) Group T, intense presence of tissue neoformation circulating lamellar birefringent eosinophilic material (arrow). (b)Group C, presence of discreet tissue neoformation (arrow).

Microscopic evaluation showed the presence of neoformed tissue, composed by connective tissue associated with immature bone tissue. In the T group, it was possible to observe that this tissue neoformation circumscribed a focal area containing lamellar birefringent eosinophilic material, which was associated with the implant, exhibiting blood vessels and a mild inflammatory reaction signaled by the activity of mononucleated cells (Figure 1).

The microscopic findings were similar in both groups, and a difference in the amount of neoformed tissue was observed, which was confirmed after the morphometric analysis, which revealed a significant difference in the amount of neoformed tissue at the bone/implant interface of the operated limb.

The stimulus of tissue neoformation observed in the present study is directly related due to the effect of using implants, and this beneficial effect is also reported in many studies with other biomaterials as well. The composite of chitosan, gelatin, and PRP brings many advantages when used with hydroxyapatite [32], which suggests that these materials have superior results when used in association when compared to defects without implants.

When comparing isolated uses and the association of biomaterials such as chitosan and PRP, the use of the combination shows superior effects when compared to its isolated use [33], suggesting a potentiation relationship since these biomaterials tend to stimulate different phases of the healing of bone and/or cartilage tissue.

The same happens when we observed the effect on healing after the addition of ceramics such as hydroxyapatite, in biomaterial implant formulations for bone and/or cartilage healing [34], which, although acting in more specific phases of the healing of these tissues, potentiates the healing of bone tissue.

Nevertheless, in the present study, no comparisons were made between the individual use of the biomaterials, not being possible to affirm that the effect of the association is superior to the isolated use of them.

## 4. Conclusions

In view of the observed, it is possible to conclude that the composite based on the association of chitosan, hydroxyapatite, and platelet-rich plasma favored bone and cartilaginous healing.

## Figures and Tables

**Table 1:** Clinical-orthopedic evaluation of rabbits submitted to trochlear osteotomy.

Animal	Lameness	Painful reaction	Muscle atrophy
1	Absent	Absent	Absent
2	Absent	Absent	Absent
3	Absent	Absent	Absent
4	Treatment limb	Absent	Absent
5	Absent	Absent	Absent
6	Absent	Absent	Absent
7	Absent	Absent	Absent
8	Control limb	Absent	Absent
9	Absent	Absent	Absent
10	Absent	Absent	Absent
11	Absent	Absent	Absent
12	Treatment limb	Absent	Absent

**Table 2:** Evaluation of subchondral bone healing reaction index based on radiographs of both limbs (groups) of the animals.

	Pelvic limb	
	Right (group C)	Left (group T)
Absence of bone reaction	3/12 (25%)	4/12 (33%)
Presence of moderate bone reaction	8/12 (67%)	5/12 (42%)
Presence of intense bone reaction	1/12 (8%)	3/12 (25%)

**Table 3:** Mean and standard deviation of the area in *μ*m^2^ obtained through histomorphometry of the newly formed tissue at the bone/implant interface in the femoral trochlea of rabbits.

	Pelvic limb	
	Right (group C)	Left (group T)
Mean	14.779,15b	24.476,15a
Standard deviation	5.294,18	10.363,14

Means followed by diverse letters on the same line present statistical difference (*p* < 0.05) by the Kruskal–Wallis test.

## Data Availability

The data used to support the findings of this study are available from the corresponding author upon request.
